# Resignation of Prof. John Bell

**Published:** 1852-03

**Authors:** 


### RESIGNATION OF PROF. JOHN BELL.

Dr. Bell, the learned professor of Theory and Practice in the Medical College ef Ohio, has resigned his chair, and returns to Philadelphia at the close of the present term. We regret to make this announcement; we had hoped that Dr. Bell had come to reside permanently among us. His ripe scholarship, profound medical learning, cultivated taste, and estimable social and moral qualities render him an acquisition and an ornament to any society; and he will carry back with him to his former home the good wishes and regards of all whose acquaintance he has made during his brief sojourn in the West.

